# Adipose-Derived Extracellular Vesicles: Systemic Messengers and Metabolic Regulators in Health and Disease

**DOI:** 10.3389/fphys.2022.837001

**Published:** 2022-02-23

**Authors:** Simon T. Bond, Anna C. Calkin, Brian G. Drew

**Affiliations:** ^1^Baker Heart and Diabetes Institute, Melbourne, VIC, Australia; ^2^Central Clinical School, Monash University, Melbourne, VIC, Australia; ^3^Baker Department of Cardiometabolic Health, University of Melbourne, Melbourne, VIC, Australia

**Keywords:** adipose tissue, extracellular vescicles, exosome, metabolic homeostasis, white adipose tissue, brown adipose tissue, adipose tissue secretome

## Abstract

Adipose tissue is comprised of a heterogeneous population of cells that co-operate to perform diverse physiological roles including endocrine-related functions. The endocrine role of adipose tissue enables it to communicate nutritional and health cues to other organs, such as the liver, muscle, and brain, in order to regulate appetite and whole body metabolism. Adipose tissue dysfunction, which is often observed in obesity, is associated with changes in the adipose secretome, which can subsequently contribute to disease pathology. Indeed, secreted bioactive factors released from adipose tissue contribute to metabolic homeostasis and likely play a causal role in disease; however, what constitutes the entirety of the adipose tissue secretome is still poorly understood. Recent advances in nanotechnology have advanced this field substantially and have led to the identification of small, secreted particles known as extracellular vesicles (EVs). These small nano-sized lipid envelopes are released by most cell types and are capable of systemically delivering bioactive molecules, such as nucleic acids, proteins, and lipids. EVs interact with target cells to deliver specific cargo that can then elicit effects in various tissues throughout the body. Adipose tissue has recently been shown to secrete EVs that can communicate with the periphery to maintain metabolic homeostasis, or under certain pathological conditions, drive disease. In this review, we discuss the current landscape of adipose tissue-derived EVs, with a focus on their role in the regulation of metabolic homeostasis and disease pathology.

## Introduction

Adipose tissue and its secreted factors play an important role in maintaining metabolic homeostasis. However, dysfunctional adipose tissue, which is often observed in obesity, can contribute to the development of obesity-related complications (Rosen and Spiegelman, 2006). Traditionally, white adipose tissue is described as a fat storage site and brown adipose tissue as a thermal regulator; however, adipose tissue is now known as one of the largest endocrine organs (Coelho et al., 2013). Indeed, it is only within the last fifty years that adipose tissue has been recognised as having endocrine capacity, when it was shown to secrete unknown factors that influenced appetite. These factors, now known as the hormones adiponectin and leptin, are well recognised factors secreted from adipose tissue. Leptin was one of the first adipose secreted factor discovered to play a role in appetite suppression, where humans and animals deficient in leptin exhibited hyperphagia and early onset obesity, highlighting the important endocrine role that adipose tissue plays in maintaining metabolic homeostasis (Zhang et al., 1994; Halaas et al., 1995; Pelleymounter et al., 1995). Since those initial discoveries, it has been demonstrated that adipose tissue modulates many systemic biological functions by releasing a vast array of secreted factors which constitute the adipose secretome, including hormones, free fatty acids, lipids, cytokines, nucleic acids, and more recently, extracellular vesicles.

## Extracellular Vesicles

Extracellular vesicles (EVs) are small lipid bound particles, released from almost all eukaryotic cells, that contain biological cargo including fatty acids, proteins, and nucleic acids, all of which can be transferred from the parent cell to a neighbouring or distant recipient cell (Keller et al., 2006; Yáñez-Mó et al., 2015; Shah et al., 2018; van Niel et al., 2018). Since their discovery, EVs have been shown to play a role in regulating a diverse range of physiological functions, which is mostly dictated by the composition of the secreted EVs (Mathieu et al., 2019). In turn, the composition of EVs is determined by the cell of origin (parent cell), which is further governed by the immediate characteristics or metabolic state of that parent cell.

**Figure 1:**
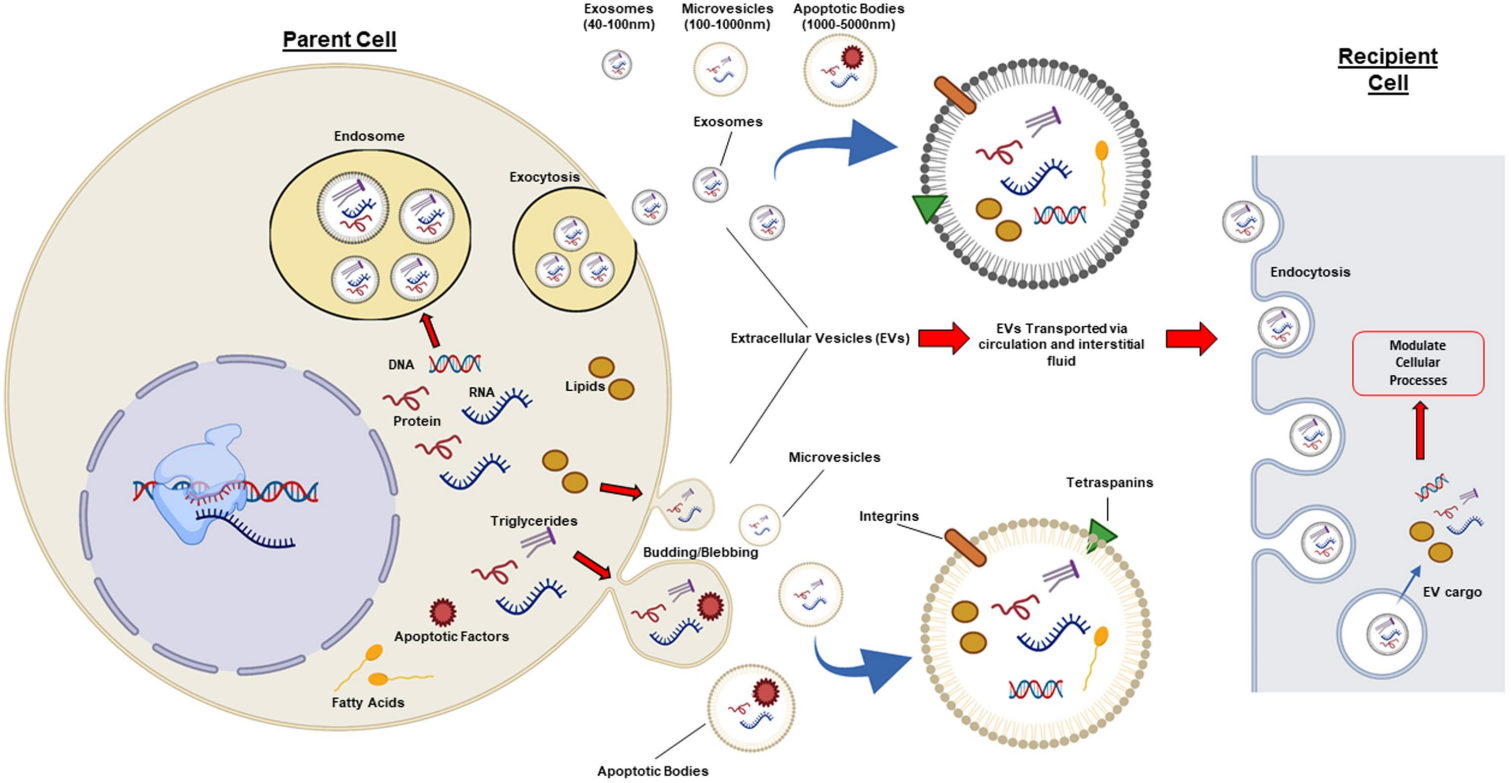
Cellular pathways involved in extracellular vesicle biogenesis. Extracellular vesicles (EVs) are a heterogeneous population of lipid bound vesicles that are released by the majority of mammalian cell types into the extracellular space and circulation. EVs consist of three main subtypes: exosomes (40–100 nm); microvesicles (100–1,000 nm); and apoptotic bodies (1,000–5,000 nm), which differ in their size, content, biogenesis, and release. Exosomes are generated and released *via* a pathway involving exocytosis, whereas microvesicles and apoptotic bodies are released *via* a budding/blebbing pathway. EV cargo includes nucleic acids, proteins, and lipids, which are highly regulated by the health status of the parent cell. Once in the extracellular space or circulation, EVs can target adjacent cells and peripheral tissues. EVs have common surface markers (tetraspanins: CD63, CD9, CD81, and CD82) and can have specific surface markers for cell targeting, such as integrins. EVs can elicit effects in recipient cells *via* cell receptor interactions or *via* absorption and release of biological cargo into the cytoplasm of the recipient cell. Figure created with BioRender.com.

There has been conjecture over the classification of EVs, as there are currently no known specific markers that define each vesicle subtype. Subsequently, there is at present, no internationally accepted definition of EVs. To date, EVs have generally been classified by size (Figure 1) and include the following subtypes: exosomes (~40–100 nm), microvesicles (~100–1,000 nm), and apoptotic bodies (~1,000–5,000 nm; Gao et al., 2017; van Niel et al., 2018). Due to the fact that exosomes and microvesicles can be similar in their biochemical properties and also overlap in size, it can often be challenging to distinguish between this highly heterogeneous population of small vesicles, hence the general term “small EVs” covers both exosomes and small microvesicles (Kowal et al., 2016; Crewe et al., 2018). However, exosomes are distinct from microvesicles and apoptotic bodies in the way they are generated and subsequently secreted. Exosomes are generated *via* an endocytic pathway, while microvesicles and apoptotic bodies are formed directly *via* blebbing of the plasma membrane (Figure 1; Borges et al., 2013). Further research is required to better understand the assembly, molecular architecture, and loading of EVs, including the underlying mechanisms which govern these pathways.

## Adipose Tissue Extracellular Vesicles

There are two distinct types of adipose tissue found in mammals: white adipose tissue (WAT) and brown adipose tissue (BAT). Further to this, adipose tissue is comprised of diverse cell populations that includes adipocytes, adipose-derived stem cells (ADSC), pre-adipocytes (adipocyte progenitors), lymphocytes, macrophages, myeloid cells, pericytes, fibroblasts, endothelial cells, and smooth muscle cells, which all play a role in maintaining and regulating metabolism and immune functions(Gimble et al., 2013; Gao et al., 2017). In addition, there are three distinct types of adipocytes, which have different primary functions: white adipocytes (storage), brown adipocytes (thermogenesis), and beige adipocytes (that can have thermogenic properties similar to brown adipocytes). The three types of adipocytes can communicate with each other in order to respond to metabolic demands and environmental stimuli, including *via* the release of EVs (Zhou et al., 2021).

EVs provide mammalian adipose tissue with an additional mode of endocrine and paracrine functional capability, with the majority of the adipose tissue secretome associated with adipocyte-derived EVs (Thomou et al., 2017; Hartwig et al., 2019). Indeed, adipose tissue-derived EVs have been shown to regulate cellular processes in both local and distant tissues, and are now considered important modulators of metabolism(Thomou et al., 2017; Ji and Guo, 2019). All three types of adipocytes (white, brown, & beige) and ADSCs have all been shown to release EVs (Ogawa et al., 2010; Chen et al., 2016; Gao et al., 2017; Connolly et al., 2018; Crewe and Scherer, 2021). These different types of adipocytes can secrete EVs with varying cargo, which likely target various cell types to differentially regulate systemic functions. Furthermore, the production and specific cargo of adipose-derived EVs have been shown to be altered under metabolic stresses, such as obesity and type 2 diabetes, and can precipitate or exacerbate disease (Eguchi et al., 2016; Gao et al., 2017; Thomou et al., 2017; Shah et al., 2018; Jafari et al., 2021). These findings suggest that EVs have the potential to serve as diagnostic biomarkers of adipose tissue health and metabolic disease (Eguchi et al., 2016; Gao et al., 2017; Thomou et al., 2017; Shah et al., 2018; Jafari et al., 2021). Accordingly, investigating the fundamental mechanisms that regulate adipose-derived EVs in health and disease could yield novel insights with regard to metabolic regulation and disease progression, and may provide new diagnostic and therapeutic opportunities.

### White Adipose Tissue Extracellular Vesicles

White adipose tissue (WAT) is the primary tissue for fat storage and release in mammals but as previously mentioned, also plays an important role in maintaining whole body metabolism. WAT-secreted EVs have garnered significant interest in recent times, particularly with regard to their ability to carry miRNAs to other tissues and regulate transcriptional pathways in target cells (Thomou et al., 2017). Although previous findings have mostly focused on the miRNA harboured within WAT EVs, new studies are now investigating the biological composition of WAT EVs in greater detail, as well as whole body responses to WAT EVs.

Although our understanding of WAT EV composition, release, and function is still in its infancy, ongoing research is slowly piecing together an intricate and highly regulated signalling network. This includes the transfer of biological cargo as a means of communication within adipose tissue, between adipocytes and other adipose-resident cell types such as endothelial cells and macrophages, as well as to peripheral tissues such as the brain and liver. A significant finding in regard to WAT EV communication within adipose tissue depots was the discovery that adipose tissue-resident endothelial cells communicated and delivered active biomolecules *via* EVs to adipocytes (Crewe et al., 2018). This was elegantly demonstrated by Crewe et al. (2018) in adipose-specific Cav1 KO mice, which unexpectedly still exhibited an abundance of Cav1 in white adipocytes where the gene had been genetically ablated. This study demonstrated that white adipocytes communicate with resident endothelial cells *via* an EV mechanism, which initiates the trafficking of Cav1 from neighbouring endothelial cells to adipocytes (Crewe et al., 2018). Such findings highlight the importance of EVs to communicate between cells, which can facilitate the transfer of enzymes and other important biological cargo from one cell type to another, which is critically necessary if a recipient cell type is depleted or cannot produce a given protein/substrate themselves. Interestingly, Crewe et al. further identified that WAT EV secretion was regulated by the metabolic status of the animal. Specifically, they demonstrated that fasting increased EV secretion *via* a glucagon-stimulated pathway, a process that was almost completely blocked in genetic and diet-induced mouse models of obesity (Crewe et al., 2018). This demonstrates that adipose tissue EVs alter their function in response to changes in systemic nutrient availability. In addition to this, Crewe et al. (2021) have demonstrated that the cargo of WAT-derived EVs is altered in response to whole body nutritional status. Notably, this study and many others that have investigated the proteomic cargo of WAT-derived EVs, have observed the presence of mitochondrial proteins (Lee et al., 2015; Durcin et al., 2017; Crewe et al., 2018; Hartwig et al., 2019; Clement et al., 2020). Indeed, WAT EVs package various aspects of mitochondrial cargo based on nutritional state. For example, WAT EVs from fasted mice contain elevated levels of electron transport chain proteins and reduced levels of mitochondrial fatty acid oxidation proteins (Crewe et al., 2018). In the setting of obesity, stressed white adipocytes release EVs that contain functional but oxidatively damaged mitochondrial components (Crewe et al., 2021). Together, these findings further support the notion that the generation of EVs, and their composition, is regulated by whole body nutritional state. The ability of WAT to selectively sort EV cargo based on nutritional or pathological state has significant implications regarding tissue cross-talk and disease, which could provide new avenues for the treatment of obesity and metabolic disease.

In addition to the exchange of EVs with endothelial cells, WAT EVs can also communicate with other-resident cell types located within the stromal vascular fraction of adipose tissue. WAT EVs reciprocally communicate with adipose tissue macrophages (ATMs) to regulate inflammatory pathways and insulin sensitivity, and vice versa (Kranendonk et al., 2014). Specifically, WAT EVs can stimulate the differentiation of monocytes into macrophages and promote inflammatory actions leading to insulin resistance (Kranendonk et al., 2014). Moreover, when ATM EVs isolated from healthy or obese mice were administered *in vivo*, they had profound systemic effects. When EVs from ATMs of obese mice were administered to lean, healthy mice, this induced insulin resistance in adipocytes, myocytes, and hepatocytes (Ying et al., 2017). Conversely, EVs isolated from ATMs of healthy mice improved insulin sensitivity when administered to obese mice (Ying et al., 2017; Liu et al., 2019). Similar effects were observed with EVs derived from white adipose tissue of genetically obese *ob/ob* mice, which also led to insulin resistance when administered to healthy mice. This phenomenon was reduced in TLR4 KO mice, suggesting that WAT EVs communicate with immune cells *via* this receptor to impact on insulin sensitivity and in turn, alter glucose metabolism (Deng et al., 2009; Gao et al., 2017). The relationship between white adipocytes and ATMs has a strong influence on metabolic homeostasis, adipose tissue function, and disease progression. Thus, the communication between adipocytes and ATMs *via* EVs requires further investigation to reveal the processes that regulate this reciprocal communication and to understand the conditions that lead to different physiological and pathological outcomes.

### Visceral and Subcutaneous White Adipose Tissue Extracellular Vesicles

Another factor that influences WAT EV composition and their consequent systemic effects, is the region or adipose depot from which the EVs are derived. The abundance of each different adipose tissue depot confers a different degree of cardiometabolic risk, with visceral adipose tissue (VAT) being associated with increased risk, and subcutaneous adipose tissue (SAT) associated with reduced risk (The ADIPOGen Consortium et al., 2015). The mechanisms precipitating this difference between VAT and SAT are not fully understood, but the varying effects of WAT-derived EVs from the different depots could play a role. For instance, EVs from VAT have been implicated in insulin resistance in human liver and muscle cells (Kranendonk et al., 2014). A recent key study that characterised human-derived VAT and SAT EVs isolated from morbidly obese individuals, identified 574 proteins in VAT-derived EVs, and 401 proteins in SAT-derived EVs (Camino et al., 2021). Of the 574 proteins identified in VAT, only 50% overlapped with EVs isolated from SAT (Camino et al., 2021). In addition, VAT EVs contained more obesity-related adipokines and were enriched for proteins implicated in adipose tissue inflammation and insulin resistance, demonstrating that the diversity in EV composition can be dependent upon their adipose depot of origin (Camino et al., 2021). Interestingly, the different composition of EVs from VAT and SAT could partially explain how VAT and SAT confer distinct disease risk profiles, which will be discussed in the following section.

An important consideration of EV functionality is their ability to cross the blood brain barrier. Indeed, it has been demonstrated that VAT EVs can cross the blood brain barrier and deliver cargo to the hypothalamus, where VAT EVs from obese mice were shown to increase food intake and body weight in chow fed mice (Gao et al., 2020). This was suggested to be a result of the transfer of miRNA and lncRNA packaged into VAT EVs, which were transferred to POMC neurons in the hypothalamus, subsequently increasing metastasis-associated lung adenocarcinoma transcript 1 (MALAT1) expression, and in turn activating mTORC1 signalling (Gao et al., 2020). This same mechanism has previously been shown to reduce food intake, with high-fat fed mice that were administered VAT EVs from lean mice demonstrating reduced appetite and weight gain *via* reduced mTORC1 signalling in the hypothalamus (Gao et al., 2020). Adipose EVs can also impact myocardial signalling. Specifically, EVs isolated from primary mouse epididymal adipocytes (VAT) treated with high glucose and palmitate, then injected intramyocardially 48 h prior to myocardial ischemia/reperfusion (MI/R), exacerbated MI/R injury in a mouse model of diabetes (Gan et al., 2020). This effect was attributed to the microRNA, miR-130b-3p, which was elevated in EVs from diabetic epididymal adipocytes, as well as with the suppression of cardioprotective and anti-apoptotic pathways (Gan et al., 2020). Conversely, Crewe et al. (2021) demonstrated that EVs shed from stressed adipocytes in obese mice contained components of mitochondria that had been damaged by oxidative stress. These EVs targeted cardiac tissue, where a burst of ROS was observed, which preconditioned the heart against ischemia/reperfusion injury by stimulating antioxidant signalling pathways (Crewe et al., 2021). This study demonstrated that not only can adipose EVs transport functional mitochondrial particles between tissues, but also that cargo which seemingly appeared to be toxic may instead be a “primer” to confer protection to other tissues.

Further to this effect on cardiac tissues, VAT EVs shed from pericardial (pFat) and epicardial (eFat) adipose tissues, which surround the heart, can also influence heart function (Shaihov-Teper et al., 2021). In unhealthy individuals, pFat and eFAT EVs can exacerbate existing conditions, leading to further complications. Specifically, eFat EVs from individuals with atrial fibrillation were enriched with proteins linked to distinct pro-inflammatory, pro-fibrotic, hypertrophic, and pro-arrhythmic pathways, likely contributing to the development of atrial myopathy and fibrillation (Shaihov-Teper et al., 2021). Moreover, EVs from pericardial fat (pFat) of obese mice promoted pathological vascular remodelling (Li et al., 2019). In obese mice, pFat that exhibited chronic low-grade inflammation, along with adipocyte hypertrophy and pro-inflammatory macrophage infiltration, secreted EVs which contained elevated levels of miR-221-3p (Li et al., 2019). miR-221-3p has previously been found to promote white adipose tissue inflammation and impair insulin sensitivity (Peng et al., 2018). In mouse cardiac tissue, pFat EVs containing miR-221-3p, stimulated the proliferation and migration of vascular smooth muscle cells, and vascular dysfunction in the femoral artery by suppression of contractile genes in the arterial wall (Li et al., 2019). Thus, EVs shed from VAT depots under disease conditions, such as obesity, can have detrimental systemic effects that contribute to disease. Moreover, they have led to significant interest into the relatively understudied epicardial and pericardial adipose tissues, to understand how EVs from these depots may contribute to cardiovascular diseases.

Together, these studies demonstrate a role for WAT-derived EVs to exchange cellular material between different cell types, both within adipose tissue depots and in peripheral tissues, that can initiate physiological and pathological responses. However, to date, the tissues that are targeted and regulated by WAT EVs are not fully understood. In order to understand these processes in detail, a comprehensive characterisation of WAT EVs is required. In particular, analyses to determine the composition of WAT EVs and to identify the cells targeted by WAT EVs under different conditions is required, including the subsequent effects elicited in recipient cells targeted by WAT EVs.

### Brown Adipose Tissue Extracellular Vesicles

Brown adipose tissue (BAT) is a highly metabolically active form of adipose tissue that plays a key role in thermogenesis and maintaining metabolic health (Simcox et al., 2017). To date, the secretory properties of BAT have received little attention; however, recent studies have demonstrated that BAT does indeed mediate important endocrine functions (Keller et al., 2006; Shah et al., 2018). Although BAT adipokines (batokines) have been identified, a comprehensive understanding of batokines and the BAT secretome, including BAT EVs, is limited. Human BAT has indeed been shown to secrete EVs; however, the role of these BAT-derived EVs is poorly understood (Chen et al., 2016). Importantly, the activation of BAT results in significant changes to the number of EVs released into the circulation. For example, human BAT exhibits a 9-fold increase in the secretion of EVs following cold-induced thermogenesis, one of the primary functions of BAT, suggesting that EV release is likely to be critical to this BAT activity (Chen et al., 2016; Thomou et al., 2017). It remains unclear why adrenergic stimulation increases BAT EV secretion, and even more so, details of the molecular characteristics and physiological role of this pathway and of these EVs (Chen et al., 2016).

In light of these findings, there has been renewed interest into BAT, in particular BAT EVs and their therapeutic potential to mimic activated BAT for the treatment of obesity. For example, BAT EVs released from *ex vivo* sections of mouse BAT tissue elicited beneficial effects in high-fat diet fed mice, by improving glucose tolerance and hepatic steatosis (Zhou et al., 2020). While supportive of a beneficial outcome, further work needs to be performed to repeat these studies at thermoneutrality and to determine the composition and target tissues/cells of these BAT EVs (Zhou et al., 2020). In addition, the components of BAT EVs that were responsible for the beneficial health effects observed are still unknown. To address this, a study by Scheele et al. investigated the proteome of conditioned media from human primary BAT-like adipocytes and identified and validated the protein, ependymin-related protein 1 (EPDR1), as a novel batokine (Deshmukh et al., 2019). EPDR1 promoted the differentiation of pre-adipocytes into brown or beige adipocytes (Deshmukh et al., 2019). When recombinant EPDR1 was subcutaneously injected into mice, it led to beneficial metabolic effects, such as increased energy expenditure, likely through programming of pre-adipocytes to differentiate into thermogenic adipocytes (Deshmukh et al., 2019). Conversely, reduced oxygen consumption and physical activity were observed in EPDR1 KO mice (Deshmukh et al., 2019). To confirm the role of EPDR1 as a batokine and in adipose tissue biology, it would be pertinent to investigate this further using tissue-specific animal models, such as EPDR1 overexpression or deletion specifically in WAT or BAT. Furthermore, it remains to be determined whether EPDR1 is packaged into BAT EVs, although from the secreted proteins, this study identified; a 97% overlap with Vesiclepedia and an 80% overlap with ExoCarta suggesting that the majority of secreted BAT proteins is indeed packaged into EVs (Deshmukh et al., 2019).

Although there is a lack of knowledge in regard to the composition of BAT EVs, similar to EVs released by WAT, BAT EVs have been shown to regulate the function of other tissues, such as the liver, through the transport of miRNA (Thomou et al., 2017). Specifically, BAT EVs can regulate fibroblast growth factor 21 (FGF21) levels in the liver *via* packaged miRNAs originating from BAT (Thomou et al., 2017). This was demonstrated in adipose-specific (white and brown) Dicer KO mice, which lack the machinery to correctly process miRNAs, leading to adipose tissue devoid of functional miRNA and a subsequent decline in the levels of circulating exosomal miRNAs (Thomou et al., 2017). Dicer KO mice developed aspects of lipodystrophy, BAT whitening, and insulin resistance (Thomou et al., 2017). This phenotype was rescued *via* the transplantation of wild-type mouse BAT into Dicer KO mice, which restored the levels of circulating exosomal miRNA, leading to improved glucose tolerance and reduced fibroblast growth factor-21 (FGF21) in liver and serum (Thomou et al., 2017). Such findings demonstrate that BAT EVs constitute an important source of exosomal miRNAs in the circulation.

These novel findings further enforce the important role of BAT, and BAT EVs, in the maintenance of metabolic health. As a result, BAT EVs are now emerging as an exciting and novel therapeutic modality for the treatment of metabolic disease. BAT EVs could improve complications associated with obesity and impaired metabolism and may offer a realistic therapeutic avenue to harness the metabolic benefits of activated BAT. This is exemplified by the fact that BAT activators have failed to translate to the clinic, as BAT is often reduced or dysfunctional in obesity. In addition, the tissue-specific delivery of small chemical activators remains a significant challenge. Moving forward, rather than activating BAT, it could be plausible to isolate EVs *ex vivo* from healthy activated BAT and administer these to obese individuals to mimic the beneficial effects of BAT activation. BAT EVs could thus act to recapitulate the metabolic benefits of activating healthy BAT in the setting of obesity, by targeting and modulating other metabolic tissues to increase energy expenditure and browning of WAT.

### Beige Adipocyte Extracellular Vesicles

Beige (or brite) adipocytes are an intermediate adipocyte subtype, that are dispersed throughout adipose tissues, and display thermogenic properties (Wu et al., 2012). Beige adipocytes are proposed to have a high cellular plasticity, which provides the ability of the beige adipocyte to switch from white adipocyte-like functions to brown adipocyte-like functions as required, thus making them distinct from both white and brown adipocytes. Beige adipocytes have been identified in adult humans and have been shown to release EVs (Wu et al., 2012; Chen et al., 2016). Similar to BAT EVs, beige adipocytes increase the release of EVs when activated (Chen et al., 2016). For example, in response to cAMP, there was an 11-fold increase in EV release from beige adipocytes, which was not observed in white or brown adipocytes (Chen et al., 2016). EVs released from these activated beige adipocytes contained factors that were protective against diabetes, which when administered to primary white adipocytes, increased insulin sensitivity, and insulin-stimulated glucose uptake (Su et al., 2018).

Currently, beige adipocyte EVs remain largely under-investigated, but similar to BAT-derived EVs, hold significant therapeutic potential for the treatment of obesity. Of specific interest is the possibility that EVs released from beige and brown adipocytes might perpetuate further beiging and activation of BAT pathways in WAT and adipocyte progenitors. However, several limitations remain regarding the interrogation of beige adipocytes, particularly regarding the difficulty in distinguishing beige cells from white and brown adipocytes and in addition, obtaining sufficient quantities of beige adipocytes. However, new *in vitro* techniques using human-induced pluripotent stem cells (iPSC) have yielded promising results, yet the study of beige adipocyte EVs *in vivo* remains challenging (Su et al., 2018).

### Adipose-Derived Stem Cell Extracellular Vesicles

Adipose tissue is a rich source of mesenchymal stem cells (MSCs), commonly referred to as adipose-derived stem cells (ADSCs). Much like other cell types, new insights have revealed that ADSCs secrete EVs that exert protective effects in several disease settings, with the ability to regulate adipocyte progenitor differentiation and adipocyte function (Zhao et al., 2018; Li et al., 2020; An et al., 2021). Specifically, ADSC-derived EVs can attenuate the progression of obesity-related complications *via* effects on WAT inflammation, systemic insulin resistance, dyslipidemia, and hepatic steatosis in diet-induced mouse models of obesity (Zhao et al., 2018). Improvements in these parameters were attributed to activation of M2 (pro-resolving) macrophage polarisation and beiging of WAT (Zhao et al., 2018). These findings have since been recapitulated by EVs isolated from human ADSCs during either white or beige adipogenic differentiation (Jung et al., 2020). EVs isolated from differentiating ADSCs induced cell reprogramming by promoting the differentiation of independent ADSCs into either white or beige adipocytes, respectively (Jung et al., 2020). Moreover, ADSC EVs released during beige adipogenic differentiation were administered to high-fat diet fed mice, which was associated with an amelioration of hepatic steatosis and glucose tolerance, thought to be mediated by miRNAs within the EVs (Jung et al., 2020).

Currently, the characterisation and therapeutic potential of ADSC-derived EVs from different adipose depots, and their regulation in different disease pathologies, needs to be further explored. Future studies could utilise the therapeutic potential of ADSC EVs to complement, or even replace, current stem cell therapies. The ability to easily isolate human ADSC and culture them allows researchers to readily isolate EVs from ADSCs exposed to various treatments for characterisation and assessment of their physiological effects.

## Adipose Tissue Extracellular Vesicles in Disease

**Figure 2:**
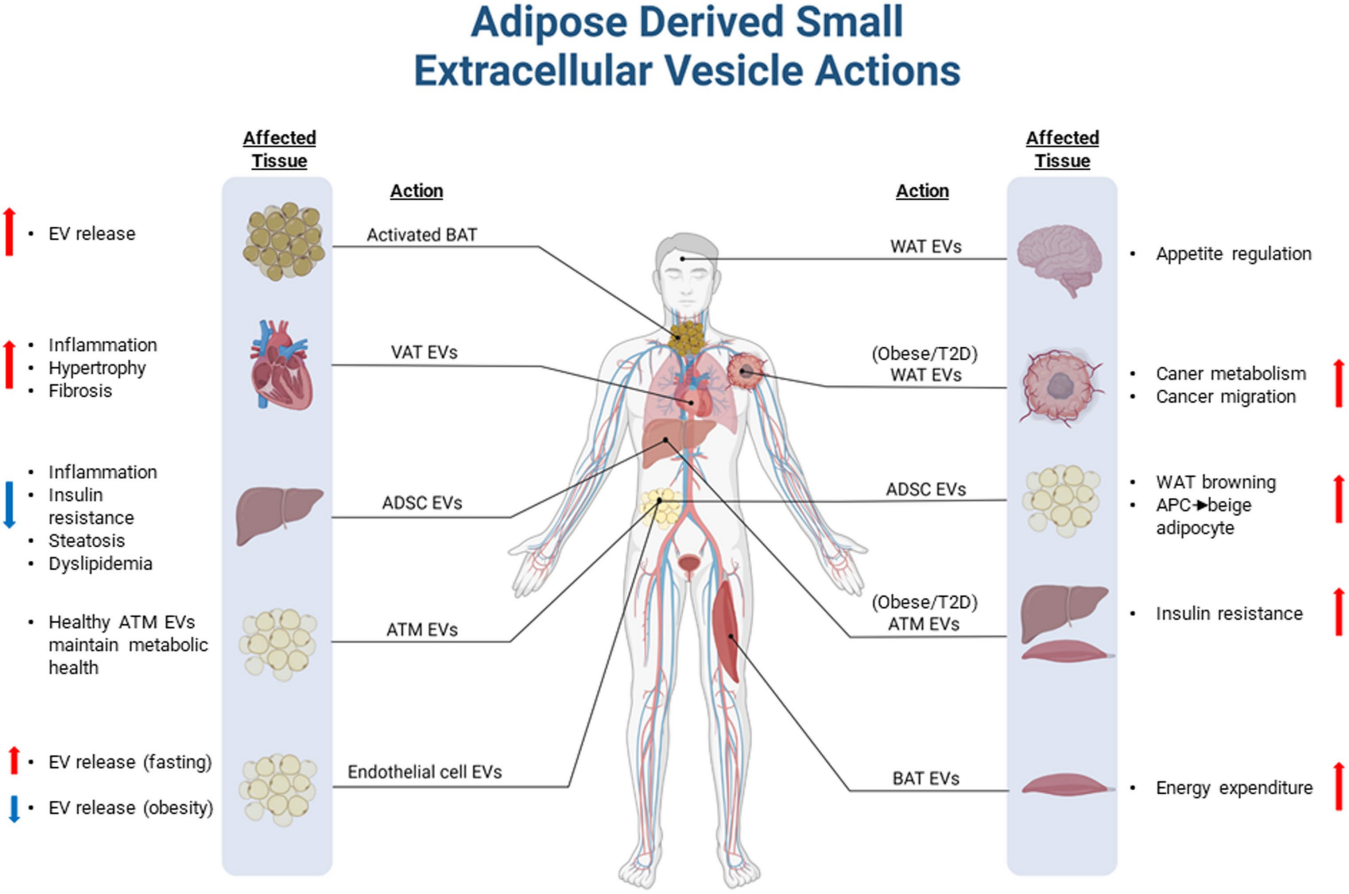
Overview of adipose-derived small extracellular vesicles in health and disease. Extracellular vesicles (EVs) derived and released from adipose tissues can modulate physiological processes in peripheral tissues. In healthy individuals, adipose EVs may play an important endocrine and paracrine role to maintain metabolic homeostasis through reciprocal communication with peripheral tissues and other cell types residing in adipose tissue depots. EVs shed from unhealthy adipose tissue, which often occurs in diseases such as obesity and type 2 diabetes, can exacerbate or drive pathologies associated with disease complications. VAT, Visceral adipose tissue; EVs, Extracellular vesicle; ATM, Adipose tissue macrophage; WAT, White adipose tissue; BAT, Brown adipose tissue; APC, Adipocyte progenitor cell; ADSC, Adipose-derived stem cell; and T2D, Type 2 Diabetes. Figure created with BioRender.com.

Research to date has demonstrated that EVs isolated from resident cells of healthy adipose tissue including adipocytes, macrophages, and stem cells have the ability to improve metabolic complications (Figure 2). Conversely, there is sufficient evidence to demonstrate that EVs released from unhealthy adipose tissue, such as in the setting of obesity, can contribute to disease pathologies. Metabolic diseases are inherently complex conditions, which is partly explained by the multiple tissues involved and the intricate cross-talk that occurs between these tissues under various pathological and environmental conditions. Understanding the roles that EVs play in this complex interplay, particularly in regard to adipose tissue, may help us to uncover novel pathological drivers of disease.

Although EVs are released from cells in a constitutive manner, pathophysiological stimuli can modulate EV biogenesis and release. Furthermore, protein and miRNA packaging into EVs can be selective under conditions of physiological change or pathological insults, thus mirroring the microenvironment in the parent cell (Keller et al., 2006; Shah et al., 2018; Mathieu et al., 2019). Garcia-Martin et al. (2021) recently described a mechanism by which cells actively sort miRNA for cellular retention or packaging into EVs *via* miRNA motifs. These findings can provide clues to assist with identifying the tissue of origin for circulating EVs, as well as provide novel approaches for RNA-mediated therapies. However, little is known about how EV cargo is regulated in response to disease. In the setting of obesity, an increase in circulating adipose-derived EVs is observed, where they are implicated in the development of obesity-associated metabolic disorders including insulin resistance and type 2 diabetes (Gao et al., 2017; Kwan et al., 2021). Some of the pathways that are regulated by adipose-derived EVs in obesity include the stimulation of monocyte differentiation and macrophage activation, upregulation of tumour necrosis factor-α (TNF-α) and interleukin-6 (IL-6), and inflammation and dysregulation of the transforming growth factor-beta (TGF-β) pathway which progresses the development of fatty liver disease (Deng et al., 2009; Koeck et al., 2014). Many of these factors have been shown to be regulated through transcriptional reprogramming *via* the transfer of miRNAs that are packaged into adipose-derived EVs. This is observed in EVs isolated from VAT of obese individuals, which contributed to hepatic and skeletal muscle insulin resistance mediated by adipocyte-derived miR-27a inhibition of Protein kinase B (Akt) phosphorylation and peroxisome proliferator activated receptor alpha (PPARα) expression (Kranendonk et al., 2014; Yu et al., 2018).

In addition to the negative metabolic roles of adipose-derived EVs described in the setting of metabolic syndrome, there is now sufficient evidence that EVs shed from unhealthy adipose tissue can also exacerbate other conditions, such as cancer (Figure 2). Adipose EVs released under certain conditions, such as in the setting of obesity or type 2 diabetes, can regulate and sustain the high energy demands of cancer cells (Jafari et al., 2021). This is facilitated by the transfer of nucleic acids which can upregulate metabolism in cancer cells, or by the transfer of metabolic substrates and machinery (Lazar et al., 2016; Clement et al., 2020; Jafari et al., 2021). White adipocytes have been shown to package fatty acids and fatty acid oxidation enzymes into EVs for delivery to tumours, providing cancer cells with the necessary substrates and machinery to facilitate fatty acid oxidation (Lazar et al., 2016; Clement et al., 2020). Importantly, EVs shed from unhealthy adipose tissue can increase the aggressiveness of cancers by not only enhancing metabolism, but also by facilitating the migration of cancer cells *via* induction of the epithelial-to-mesenchymal transition process (Lazar et al., 2016; Jafari et al., 2021). This raises the possibility of targeting adipose EVs as a potential treatment for cancer. In addition, adipose EVs may become reliable markers of cancer severity or progression. While current research relating to adipose-derived EVs is promising in regard to disease development and progression, the mechanisms and key mediators of these processes remain poorly understood.

### Therapeutic Considerations

Extracellular vesicles are emerging as an exciting new tool with diagnostic and therapeutic potential for a variety of diseases including metabolic diseases and cancer (Ghafouri-Fard et al., 2021). The stability of EVs compared with other therapeutics is a favourable aspect where for instance, EVs isolated from ADSCs are easily stored for long periods, compared with more traditional ADSC therapeutics. In addition, EV dosage can be easily controlled, and to date, there is no evidence of rejection by the recipient’s immune system (An et al., 2021). Further to this, with the potential for targeted delivery of EV-encapsulated cargo, recent technological advances are leading to large scale commercial production of EVs (Whitford and Guterstam, 2019). This includes the production of “näive” or native EVs (naturally produced by cells) and also the production of engineered EVs loaded with specific cargo. Even though there are currently no FDA approved EV therapeutics, several pharmaceutical companies and institutions have now commenced clinical trials using EVs (Fuster-Matanzo et al., 2015; Chen et al., 2020). Clinical trials are currently utilising EVs from a variety of sources and for a diverse range of purposes, such as cancer treatment, wound healing, disease biomarkers, and vaccines, which to date, have been well tolerated, and are yielding positive results (Fuster-Matanzo et al., 2015; Santos and Almeida, 2021). Given this, many companies are now investing in new technologies so that EV production can be readily up scaled for commercial purposes (Chen et al., 2020; Santos and Almeida, 2021).

Other therapeutic opportunities could include the identification of factors that inhibit the biogenesis, release, or delivery and function of EVs released from unhealthy adipose tissue. By targeting these pathways, it could be possible to prevent the detrimental effects mediated by unhealthy adipose tissue EVs. Further investigation into the fundamental biology of adipose tissue-derived EVs, in health and disease, will help to progress the translation of clinical applications which can leverage off the mechanisms that underpin adipose EV biology.

## Conclusion

New research into EV biology has provided us a better understanding of how adipose tissue-derived EVs can modulate metabolism within adipose tissue and other peripheral organs. With technical advances in isolation, purification, and characterisation of EVs, we are gaining a clearer understanding of the roles of adipose-derived EVs in both health and disease. It is now well established that the communication between adipose tissue and other organs *via* secreted factors, including those in EVs, is essential for preserving metabolic health. However, many questions still remain in this field, including the need to understand how these particles communicate with each other and other tissues. In particular, what are the differences in EV composition and function from the various adipocytes and the different adipose regions? In addition to this, it is unclear how recipient cells distinguish between the EVs they receive and co-ordinate their biological responses. In order to answer these questions, it is essential to develop appropriate laboratory tools to investigate adipose EVs. To this end, we need to understand whether there are differences in EV composition between primary and immortalised cell types, and whether they faithfully recapitulate adipose tissue EVs *in vivo*. It is possible that novel animal models could be developed to interrogate the role of EVs in a tissue-specific manner, which would allow better clarity around the quantity, destination, and effects of EVs from specific tissues. Overall, while the EV field is an exciting and emerging area, significantly more work is necessary to understand the fundamental biology of EV function before we can ultimately harness their potential for therapeutic utility.

## Author Contributions

BD and SB directed the concepts in the review and wrote the manuscript. AC contributed to discussion and provided the critical review. All authors contributed to the article and approved the submitted version.

## Funding

We acknowledge funding support from the Victorian State Government OIS program to Baker Heart & Diabetes Institute. BD and AC received support from the National Heart Foundation of Australia, Future Leader Fellowship scheme (101789 and 105631, respectively). Aspects of this review were supported by a grant from the Australian National Health and Medical Research Council to BD (NHMRC, APP1128060).

## Conflict of Interest

The authors declare that the research was conducted in the absence of any commercial or financial relationships that could be construed as a potential conflict of interest.

## Publisher’s Note

All claims expressed in this article are solely those of the authors and do not necessarily represent those of their affiliated organizations, or those of the publisher, the editors and the reviewers. Any product that may be evaluated in this article, or claim that may be made by its manufacturer, is not guaranteed or endorsed by the publisher.
